# Clinical 7 Tesla magnetic resonance imaging: Impact and patient value in neurological disorders

**DOI:** 10.1111/joim.20059

**Published:** 2025-01-08

**Authors:** Elisabeth de Vries, Caroline Hagbohm, Russell Ouellette, Tobias Granberg

**Affiliations:** ^1^ Department of Neuroradiology Karolinska University Hospital Stockholm Sweden; ^2^ Department of Clinical Neuroscience Karolinska Institutet Stockholm Sweden

**Keywords:** dementia, diagnosis, multiple sclerosis, neurology, radiology, vascular disease

## Abstract

Magnetic resonance imaging (MRI) is a cornerstone of non‐invasive diagnostics and treatment monitoring, particularly for diseases of the central nervous system. Although 1.5‐ and 3 Tesla (T) field strengths remain the clinical standard, the advent of 7 T MRI represents a transformative step forward, offering superior spatial resolution, contrast, and sensitivity for visualizing neuroanatomy, metabolism, and function. Recent innovations, including parallel transmission and deep learning–based reconstruction, have resolved many prior technical challenges of 7 T MRI, enabling its routine clinical use. This review examines the diagnostic impact, patient value, and practical considerations of 7 T MRI, emphasizing its role in facilitating earlier diagnoses and improving care in conditions, such as amyotrophic lateral sclerosis (ALS), epilepsy, multiple sclerosis (MS), dementia, parkinsonism, tumors, and vascular diseases. Based on insights from over 1200 clinical scans with a second‐generation 7 T system, the review highlights disease‐specific biomarkers such as the motor band sign in ALS and the new diagnostic markers in MS, the central vein sign, and paramagnetic rim lesions. The unparalleled ability of 7 T MRI to study neurological diseases ex vivo at ultra‐high resolution is also explored, offering new opportunities to understand pathophysiology and identify novel treatment targets. Additionally, the review provides a clinical perspective on patient handling and safety considerations, addressing challenges and practicalities associated with clinical 7 T MRI. By bridging research and clinical practice, 7 T MRI has the potential to redefine neuroimaging and advance the understanding and management of complex neurological disorders.

## Graphical abstract

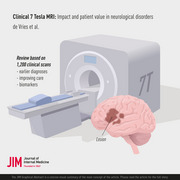

**Abbreviations**
ALS: amyotrophic lateral sclerosis
ARIA: amyloid‐related imaging abnormalities
AVMs: arteriovenous malformations
CAA: cerebral amyloid angiopathy
CEST: chemical exchange saturation transfer
CSF: cerebrospinal fluid
DWI: diffusion‐weighted imaging
FCD: focal cortical dysplasias
MRI: magnetic resonance imaging
MS: multiple sclerosis
MSA: multiple system atrophy
PD: Parkinson's disease
PRLs: paramagnetic rim lesions
PSP: progressive supranuclear palsy
QSM: quantitative susceptibility mapping
SNpc: substantia nigra pars compacta
SWI: susceptibility‐weighted imaging
T: Tesla

## Introduction to clinical 7 Tesla MRI: a new frontier in neuroimaging

The field of neuroimaging has witnessed remarkable advancements over the past few decades. Magnetic resonance imaging (MRI) has become the gold standard for visualizing the complex structures and functions of the human central nervous system without using ionizing radiation. MRI with conventional field strengths of 1.5 and 3 Tesla (T) has thereby become a cornerstone for diagnosing many neurological disorders.

**Fig. 1:**
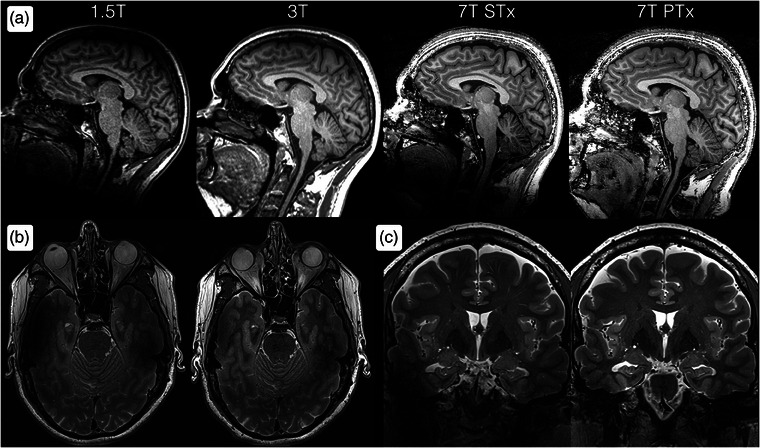
(a) Comparative acquisitions of sagittal 3D T1‐weighted imaging at 1.5 and 3 T (1.0 mm isotropic resolution) as well as 7 T (0.7 mm isotropic resolution), from left to right. The lower signal at 1.5 T manifests as more noisy images. Meanwhile, high signal can be retained at 7 T, despite higher spatial resolution relative to 3 T. The 7 T acquisitions have been performed with both conventional single transmission (STx) and the more novel parallel transmission (PTx) technologies. PTx provides more homogeneous image intensities and retains the signal in the posterior fossa and spinal cord that is lost when using STx at 7 T. (b) Axial 2D T2‐weighted imaging at 7 T with STx (left) and PTx (right), showing clearly improved depiction of the temporal lobes using PTx. (c) Corresponding coronal images.

After being used as a research tool for many years, 7 T has recently also been clinically approved. This marks a significant milestone, offering potentially higher resolution (exemplified in Fig. 1) and greater pathological insight than ever before [1]. Although the advent of clinical 7 T MRI represented a diagnostic leap forward, it has faced technical, practical, and medical challenges. These have, to a large extent, been addressed by recent hardware and software advancements in a second generation of clinical 7 T MRI, addressing prior limitations and making routine clinical use feasible.

This review explores recent technical innovations in clinical 7 T MRI and the diagnostic value it brings to patient care. Based on experience from over 1200 clinical scans conducted with a second‐generation system in the first year of operation, we provide practical insights and highlight its potential to redefine diagnostics and treatment in neurological disorders.

## Technical advancements and their clinical benefit

### Clinical approval and hardware improvements

Clinical approval of 7 T MRI, achieved in 2017, marked a milestone in ultra‐high‐field neuroimaging [1]. The introduction of second‐generation systems in 2023 has addressed key challenges of earlier models, further advancing clinical adoption.

Producing 7 T magnets—over 100,000 times stronger than Earth's magnetic field—requires helium‐cooled superconducting environments and robust magnetic shielding. Modern innovations have reduced the size and weight of 7 T scanners, enabling hospital integration despite weighing over 20,000 kg, almost double that of a 3 T scanner. Notably, though, the physical dimensions are now on par with 1.5 T scanners from the 1990s, making it possible to repurpose older bays for MRI scanners in hospitals for 7 T MRI. Active shielding has also minimized fringe fields, reducing the MRI safety zone footprint.

A key challenge in clinical 7 T MRI is achieving homogenous image quality across the brain and spinal cord, especially near air‐filled structures, such as paranasal sinuses, temporal bones, and the lungs. Innovations such as parallel transmission—using multiple transmit channels—make it possible to tailor radio waves suitable for each patient [2]. This translates into more homogenous image intensities, as exemplified in Fig. 1, and is particularly valuable in, for example, presurgical evaluation of temporal lobe epilepsy and detecting infratentorial lesions in multiple sclerosis (MS).

### Software improvements

Recent software advancements have significantly streamlined the operation of 7 T MRI systems, aligning them with conventional clinical scanners. Automated shimming, slice positioning, and standardized graphical user interfaces now reduce the need for advanced application training. These improvements simplify integration into clinical workflows.

Deep learning–based tools have transformed imaging by reducing scan times while maintaining, or even enhancing, image quality [3]. Faster scans improve patient comfort, minimize motion artifacts, and boost throughput, which is critical for high‐cost systems such as 7 T MRI. Additionally, deep learning enhances resolution, contrast, and image consistency by addressing intensity variations. These innovations enable more accurate diagnoses, particularly for subtle pathologies, and support disease monitoring and treatment evaluation.

## Practical considerations

### Safety and operational considerations

The increased magnetic field strength of 7 T MRI poses no known long‐term health risks but introduces unique challenges. Scanning is typically performed in “First Level Controlled Operating Mode,” requiring medical supervision due to elevated radiofrequency and gradient fields. Specific absorption rate limits and tissue heating demand careful monitoring, often necessitating longer acquisition times and individualized adjustments for safety.

Practical limitations include the lack of testing for many implants at 7‐T, restricting its use in patients with implanted devices—relatively common among elderly patients with neurological diseases. Additionally, current head coils have a weight limit of 30 kg, precluding imaging in small children, and the absence of 7 T MRI‐compatible ventilators further limits use in pediatric or sedated patients. Clinical approval is also limited to brain imaging, as spinal cord coils remain under development. This necessitates the use of multiple scanners for combined brain and spinal cord imaging, such as in neuroinflammatory disorders.

These factors collectively underscore the importance of balancing the benefits of enhanced imaging capabilities of 7 T MRI with stringent safety measures to minimize potential risks.

### Patient experience

Seven Tesla scanners retain the conventional bore size of 60 cm, which can feel restrictive compared to newer wide‐bore designs at 0.55 (80 cm), 1.5, and 3 T (70 cm). Patients may—similarly to scanning at conventional field strengths—experience sensory effects such as tingling or light vertigo during scans, which are generally mild and transient. Educating patients about these sensations and noise levels helps improve comfort and acceptance, crucial for clinical integration.

### Workflow and training

Effective adoption of 7 T MRI into clinical workflows demands collaboration among MRI physicists, radiologists, and technologists. Pathology‐specific protocols must balance resolution with scan time, catering to diverse clinical needs. Training radiologists in interpreting 7 T images is essential, as these scans reveal details not observable at lower field strengths. Continuous vendor support and user feedback are key to refining protocols and leveraging the system's full potential.

### Financial and infrastructure considerations

The high cost of 7 T MRI systems, coupled with increased operational expenses, presents a significant barrier. Any infrastructure modifications needed at installation as well as service contracts add to the financial burden. Institutions must carefully weigh these costs against clinical benefits, ensuring sufficient patient throughput and reimbursement models to justify investment. In practice, reimbursement strategies differ among regions, and 7 T MRI often lacks specific/higher reimbursement, making it challenging to build a financial case for the scanner.

## Clinical applications of 7 T MRI in neurological disorders

Growing evidence underscores the clinical utility and cost‐effectiveness of 7 T MRI in neurological disorders. This section highlights its key clinical applications, emphasizing enhanced diagnostic accuracy and deeper pathophysiological insights.

### Amyotrophic lateral sclerosis

Amyotrophic lateral sclerosis (ALS) is a progressive and fatal neurodegenerative disorder characterized by dysfunction of both upper and lower motor neurons. Upper motor neuron pathology involves the loss of pyramidal neurons in the primary motor cortex along with axonal loss and gliosis in the corticospinal tracts, where reactive, iron‐laden microglia and macrophages are abundant [4]. Assessing upper motor neuron burden currently relies on clinical evaluation, which is often confounded by overlapping lower motor neuron degeneration. Although electrophysiological tests provide insights into lower motor neuron dysfunction, a robust in vivo imaging biomarker for upper motor neuron impairment remains an unmet need [5]. The integration of 7 T MRI into clinical practice is advancing these possibilities, significantly enhancing the qualitative and quantitative detection of motor neuron pathology [6].

**Fig. 2:**
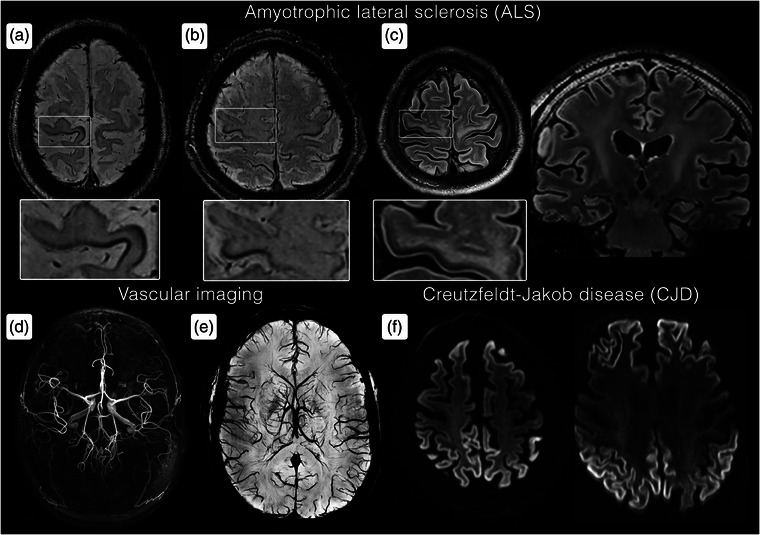
Clinical 7 T magnetic resonance imaging (MRI) acquisitions across neurodegenerative disorders and vascular applications: (a) motor band sign in the medial primary motor cortex in a person with amyotrophic lateral sclerosis (ALS) with predominantly affection of the left lower extremity; (b) motor band sign in the hand motor cortex bilaterally in a person with ALS with bilateral hand motor weakness; (c) corticospinal tract hyperintensities on T2‐weighted fluid‐attenuated inversion recovery (FLAIR) in a person with ALS; (d) time‐of‐flight (TOF) angiography without contrast agents visualizing the circle of Willis and more peripheral arteries; (e) susceptibility‐weighted imaging (SWI) minimum intensity projection (minIP) visualizing the venous brain vasculature; (f) diffusion‐weighted imaging (DWI) visualizing pathological diffusion restriction in the cortex with typical topography for Creutzfeldt–Jakob disease.

The motor band sign has emerged as a promising imaging biomarker for ALS. This rim‐like cortical hypointensity reflects iron accumulation within microglial cells in the deeper layers of the primary motor cortex, typically visualized along the posterior border of the primary motor cortex, as exemplified in Fig. 2. It is most effectively detected using T2*‐weighted imaging, susceptibility‐weighted imaging (SWI), or quantitative susceptibility mapping (QSM), which exploit the paramagnetic properties of iron to highlight these accumulations [7].

Although the motor band sign is occasionally observable with 1.5‐ and 3 T MRI [7, 8], the superior spatial resolution and increased sensitivity to field inhomogeneities of 7 T MRI allow for a more detailed characterization of iron deposition, as demonstrated both in vivo [5] and ex vivo [4]. Studies show that the motor band sign is present in a majority of patients with ALS on 7 T MRI, correlating with clinical presentation and the severity of the upper motor neuron burden [5, 9].

Another imaging biomarker in ALS is the T2 hyperintensities along the corticospinal tracts, which have been suggestive of upper motor neuron degeneration in ALS [10], with a clear depiction on 7 T in Fig. 2.

### Epilepsy

Approximately onethird of persons with epilepsy have drug‐resistant epilepsy, and among these, 30%–40% lack detectable lesions on 1.5‐ and 3 T MRI. Several studies have investigated the utility and challenges of 7 T MRI in the diagnostic work‐up of epilepsy [11], consistently demonstrating its superiority in detecting intracranial lesions or abnormalities compared to lower field strengths [12, 13, 14]. A pooled analysis reported a 31% diagnostic gain with 7 T MRI over conventional field strengths [11]. The increased signal‐to‐noise ratio at 7 T enables improved visualization of cortical components, including a more precise delineation of the gray–white matter interface. This enhancement improves the sensitivity for identifying epileptic lesions, such as focal cortical dysplasias (FCD), hippocampal sclerosis, and amygdala abnormalities [11], facilitating more accurate surgical planning and more favorable outcomes [15].

#### Focal cortical dysplasia

**Fig. 3:**
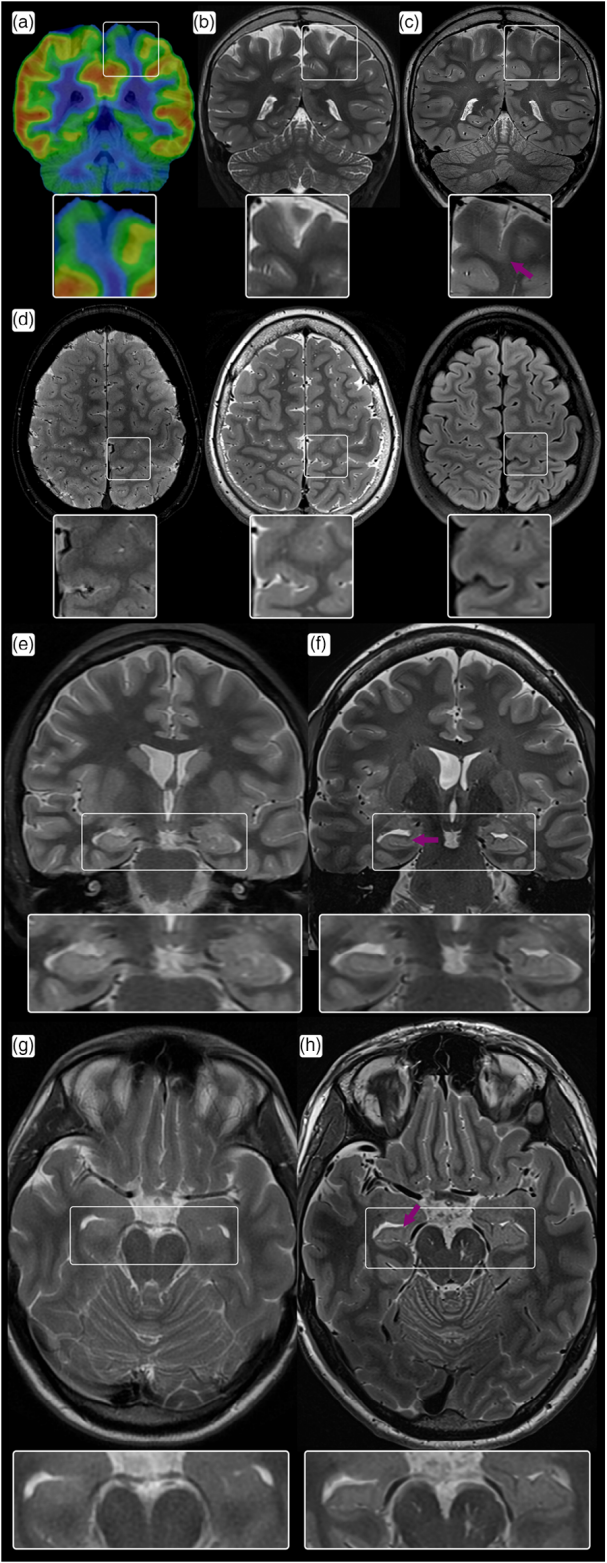
Epilepsy scanning on conventional field strengths and clinical 7 T magnetic resonance imaging (MRI) in a pediatric (a–d) and adult (e–h) patient: (a) focal glucose hypometabolism in the left parietal lobe demonstrated with PET‐MRI; (b) however, no clear anatomical correlate was seen on the corresponding anatomical coronal 2D T2‐weighted 2 mm thick slices; (c) similar imaging using a clinical 7 T scanner, providing higher in‐plane resolution and 1 mm slice thickness, showed a blurred gray–white matter boundary (arrow) consistent with a focal cortical dysplasia (FCD); (d) this finding was confirmed on axial T2*‐weighted, T2‐weighted, and fluid‐attenuated inversion recovery (FLAIR) imaging in the same 7 T scanning session; (e) coronal T2‐weighted imaging at 1.5 T reported as normal; (f) similar imaging with higher resolution with clinical 7 T MRI showing disruption of the internal architecture in the right hippocampus (arrow) as well as slightly lower size than the left; (g) corresponding axial slices on 1.5 T MRI; (h) corresponding axial slices on clinical 7 T MRI.

FCD is the most frequently resected epileptic lesion in children and the third most common in adults [16]. It involves localized cortical malformations with diverse histopathological features, classified into three subgroups (Types I–III). On MRI, FCD is characterized by increased cortical thickness, blurred gray–white matter junctions, transmantle signs, or abnormal gyral/sulcal patterns [17, 18]. Fig. 3 exemplifies FCD in a pediatric patient.

These subtle features are often overlooked on lower field‐strength MRI, particularly in lesions located deep within a sulcus. FCD is the most common etiology in patients undergoing surgery for refractory focal “non‐lesional” epilepsy, with visibility varying by subtype—Types I and IIa being the least detectable on 1.5‐ and 3 T MRI [17, 19]. However, 7 T MRI has been shown to significantly enhance diagnostic yield in these cases [13, 20].

At 7 T, thin‐slice 2D T2‐weighted imaging and 3D T1‐weighted imaging remain key techniques, with MP2RAGE being particularly useful at 7 T due to its high‐resolution imaging and ability to mitigate transmit field inhomogeneities [21, 22]. Additionally, 7 T T2*‐weighted imaging can reveal the black line sign—a distinctive intracortical hypointense band found in FCD Type IIb. Complete resection of this black line correlates with improved postsurgical seizure outcomes [19, 23].

#### Hippocampal sclerosis

Hippocampal sclerosis, the most common histopathological hallmark of temporal lobe epilepsy, involves atrophy, signal changes, and disruption of hippocampal internal architecture [24, 25]. It can be classified into four subgroups with varying prognoses for postoperative seizure control and memory function [26]. Conventional MRI primarily distinguishes hippocampal sclerosis from non‐sclerosis but provides limited detail on substructures. In contrast, 7 T MRI offers enhanced visualization of hippocampal internal architecture and enables detection of subtle pathological changes [27, 28, 29]. Fig. 3 exemplifies hippocampal sclerosis in an adult patient.

#### Polymicrogyria

Polymicrogyria—a malformation of cortical development commonly associated with epilepsy and developmental delays—benefits significantly from 7 T MRI. The improved spatial resolution at 7 T enables more detailed morphological assessments, such as visualization of dilated veins in affected areas, which are often undetectable at lower field strengths [30].

### Dementia

Dementia is caused by neurodegenerative disorders characterized by declining cognitive functions, including diseases such as Alzheimer's disease (AD), vascular dementia, Lewy body dementia, frontotemporal dementia, and less common diseases such as Creutzfeldt–Jakob disease. The most common form of dementia is AD, representing 60%–80% of all dementia types.

In clinical practice today, MRI plays a role in excluding other causes of cognitive decline as well as evaluating signs of small vessel disease, such as white matter changes, infarctions, microbleeds, and perivascular spaces, and determining the degree of cortical and hippocampal atrophy, which is typically assessed through visual inspection on 1.5‐ or 3 T MRI. Structural volumetric analysis has been used in research and clinical trials and is now increasingly used clinically. Although image inhomogeneities at higher field strengths remain a challenge, 7 T MRI and high‐resolution imaging enable more precise measurements of small anatomical regions, such as the hippocampus and subcortical structures [31].

#### Hippocampal subfield segmentations

Morphometric changes in the medial temporal lobe can serve as imaging markers for early AD due to the deposition of abnormal proteins in these regions [32]. Atrophy in the hippocampus and entorhinal cortex are markers in early AD and its prodromal stages, such as mild cognitive impairment. The hippocampus consists of several subfields—including the cornu ammonis, which is divided into four sectors, the dentate gyrus, and the subiculum. The superior spatial resolution with 7 T MRI allows for better delineation of these subfields, thus enabling the detection of subtle changes in the hippocampus [28, 33, 34]. Studies have shown that cornu ammonis 1 is one of the first regions for atrophy in AD [35]. Early detection of changes associated with AD is crucial for this patient group to initiate treatment before memory and functional decline in order to slow the progression of the disease.

#### Microbleeds

Microbleeds are defined as small round or ovoid foci of blood products, which can be detected on SWI due to the paramagnetic properties of hemosiderin. Increased magnetic field strength improves the detection of microbleeds due to enhanced susceptibility effects [36]. Seven Tesla MRI provides significantly superior detection of microbleeds compared to 3 T, increasing the detected prevalence in AD and mild cognitive impairment from 33% to 78%. The higher sensitivity of 7 T MRI can help in treatment decisions—for example, concerning the use of antithrombotic therapies in this patient group [37].

#### Cerebral amyloid angiopathy and amyloid‐related imaging abnormalities

Cerebral amyloid angiopathy (CAA) is a small vessel disease common in patients with AD, sharing similar pathomechanistic pathways involving amyloid beta deposition within the cerebral vessel walls, causing weakening of the vessels, microaneurysms, and increased risk of intracranial hemorrhage. Both diseases share the APOE ε4 allele as a common risk factor. Imaging markers for CAA include lobar microbleeds and cortical superficial siderosis, with improved detection at higher magnetic field strengths [36, 38]. Inflammatory CAA is a rare and aggressive subtype characterized by asymmetric, patchy, or confluent white matter lesions that may extend to the cortex along with multiple lobar microbleeds [39].

Monoclonal antibodies targeting amyloid beta are emerging therapies for early AD: Donanemab and lecanemab were recently approved for clinical use in the United States and have shown promising results in patients with mild cognitive impairment and early AD [40]. In November 2024, lecanemab was also approved by the European Medicines Agency [41] for treating these patients if they only have one or no copy of the APOE ε4 allele, reducing the risk of amyloid‐related imaging abnormalities (ARIA). Another risk factor for developing ARIA is CAA. ARIA is classified into ARIA‐E (edema/effusion), characterized by T2 hyperintensities that often resolve over time, and ARIA‐H (hemosiderin) with microbleeds and superficial hemosiderosis. Most ARIA cases are asymptomatic [42, 43]. However, it is crucial to identify patients with ARIA to prevent severe complications such as intracerebral hemorrhage by temporarily or permanently discontinuing the treatment. The higher sensitivity of 7 T MRI for microbleeds and siderosis may thus be translated into a more sensitive tool for monitoring these treatment‐related side effects, and it is especially useful in patients with risk factors such as CAA or the APOE ε4 allele [6, 42].

#### Creutzfeldt–Jakob disease

Creutzfeldt–Jakob disease is a rare prion disease with rapid progressive neurodegeneration. Brain MRI is often helpful in identifying these patients among memory clinic patients due to typical MRI findings, with a topography dependent on the subtype (sporadic, familial, and variant). Creutzfeldt–Jakob disease causes T2 hyperintensities in the cerebral cortex and the deep gray matter, usually the putamen and caudate nucleus, with corresponding restricted diffusion on diffusion‐weighted imaging (DWI). Often, the diffusion restriction pattern can be pathognomonic, as exemplified in Fig. 2.

### Multiple sclerosis

MS is a chronic inflammatory, demyelinating, and neurodegenerative disease of the central nervous system. It is a leading cause of neurological disability in younger adults. MS is characterized by acute inflammatory demyelinating events causing neurological symptoms with or without recovery. The MS diagnosis is based on clinical, imaging, and laboratory evidence with dissemination in time and space, with MRI being the cornerstone of diagnostics and disease monitoring [44, 45]. Increased magnetic field strength can provide better visualization of subtle pathologies and MS‐specific biomarkers, leading to earlier diagnoses and treatment, which is crucial for a better prognosis [46]. The 2024 revisions to the McDonald criteria—presented at ECTRIMS 2024 but not officially published at the time of publication of this article—put even more emphasis on radiological markers such as the central vein sign and paramagnetic rim lesions (PRLs) [47].

#### White matter lesions

The myelin loss in focal MS lesions is visualized on MRI as T2 hyperintense lesions, especially on fluid‐attenuated inversion recovery (FLAIR) sequences in which the signal from cerebrospinal fluid (CSF) is eliminated [48]. In 7 T MRI, FLAIR imaging has limitations because of increased field inhomogeneity in both the static magnetic field (B0) and the radiofrequency transmit field (B1). Therefore, other sequences—such as 3D T1‐weighted images—have a pivotal role to discriminate focal MS lesions [21, 49].

Seven Tesla MRI allows for smaller voxels, leading to enhanced spatial resolution and enabling the detection of a higher number of lesions within the white matter compared to 1.5‐ and 3 T MRI. Notably, confluent lesions on 3 T have been shown to be multiple smaller lesions using 7 T [50]. Furthermore, normal‐appearing white matter—which shows diffuse abnormalities on quantitative MRI sequences—may represent focal lesions that are not visible at lower magnetic field strengths [46].

#### Cortical lesions

For many years, MS was considered a white matter disease. With the advent of 7 T MRI in research, it has repeatedly been shown that this is not a correct categorization. In fact, both the cortex and deep gray matter are affected by focal and diffuse MS pathology. Notably, 93% of patients with MS have been shown to have cortical lesions in the early disease stages [51]. Furthermore, longitudinal 7 T scanning has shown that the lesion accrual rate is higher in the cortex than the white matter [52]. Importantly, cortical lesions are an independent predictor of future cognitive and physical impairment in MS [53, 54].

**Fig. 4:**
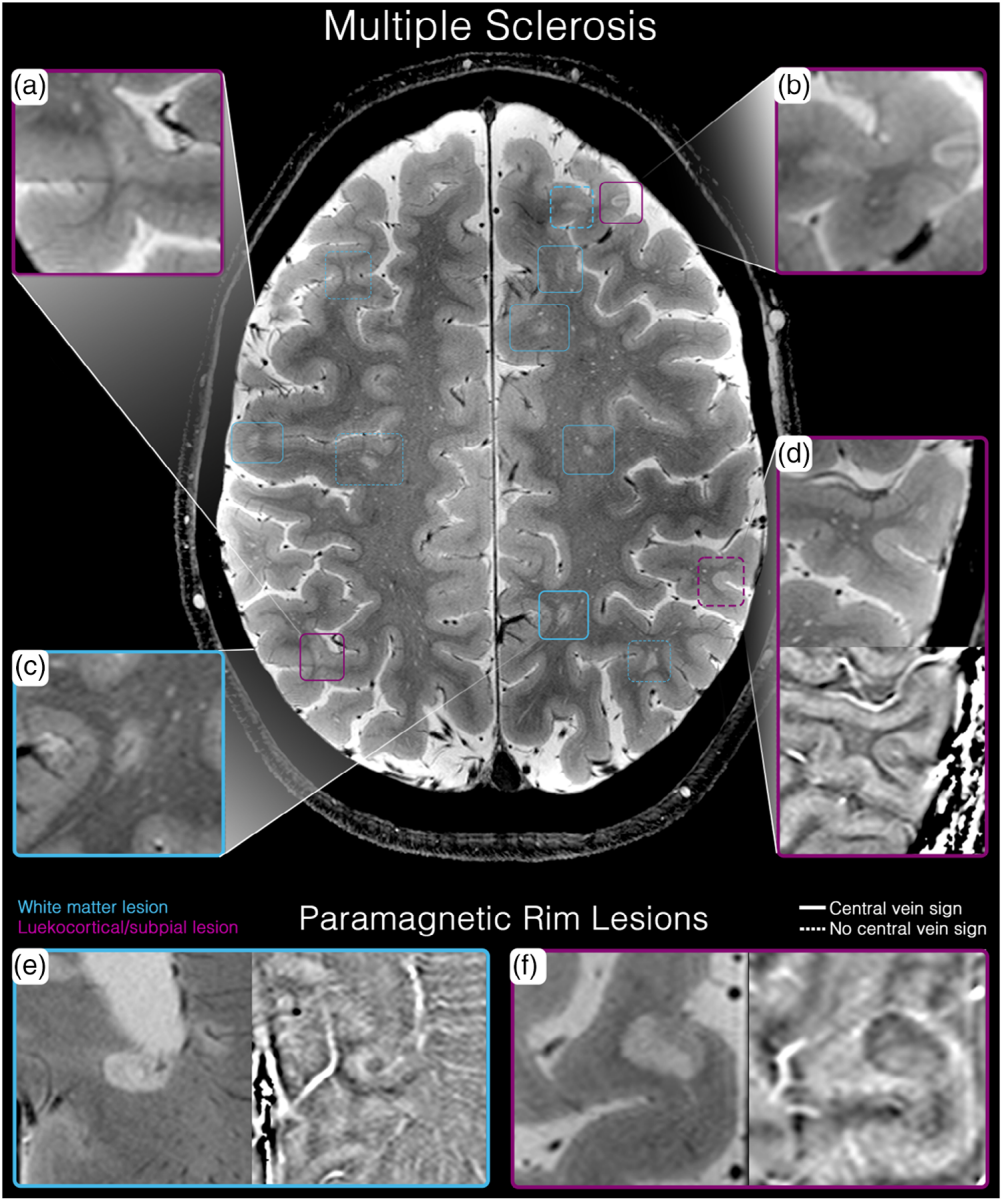
Multiple sclerosis white matter and gray matter lesions using clinical 7 T magnetic resonance imaging (MRI) (axial 2D T2*‐weighted gradient‐recalled echo with a resolution of 0.3 × 0.3 × 0.9 mm^3^: (a) leukocortical (Type I) with a central vein sign; (b) subpial (Type IV) cortical lesion with full cortical coverage and a central vein sign. (c) white matter lesion with a central vein sign; (d) subpial (Type IV) cortical lesion with full cortical coverage, a central vein sign, and a paramagnetic signature; (e) periventricular white matter lesion with a paramagnetic rim and central vein sign; (f) leukocortical (Type I) lesion with a paramagnetic signature.

Although cortical lesion detection is challenging at conventional field strengths—even with dedicated cortical imaging sequences—their detection remains an unmet clinical need because cortical lesions have high diagnostic specificity for MS and have been included in the diagnostic criteria since 2017 [44]. Due to the prevalence of MS and the importance of early diagnosis and treatment to avoid physical and cognitive dysfunction, this is perhaps the most impactful of all clinical applications of clinical 7 T MRI. Due to the thin cortical ribbon and proximity to CSF (which may obscure small cortical lesions due to partial volume effects), the higher spatial resolution and improved contrast‐to‐noise ratio of 7 T MRI provide improved detection of cortical MS lesions—especially the subpial lesions, the most common type [52, 55, 56]. For this purpose, T2*‐weighted imaging is particularly useful [57], as exemplified in Fig. 4 and Supporting Information video 1.

#### Paramagnetic rim lesions

PRLs are an emerging and specific marker for MS that were implemented in the diagnostic criteria in the 2024 revision [47]. They can be identified as hypo‐ or hyperintense rims surrounding the lesion on susceptibility‐weighted sequences, such as T2*, SWI, or QSM—with SWI being the most commonly used sequence in clinical practice [58]. The paramagnetic rim represents the accumulation of iron‐laden macrophages at the lesion edge—representing ongoing inflammation—whereas the core of the lesion is demyelinated. These findings correspond to chronic active lesions [59]. PRLs have been associated with worse clinical outcomes, higher lesion volumes, and more brain atrophy. PRLs are also more destructive compared to non‐PRLs, and the presence of PRLs has been proposed as a marker for progressive disease [60, 61, 62]. PRLs can be detected on 1.5‐ and 3 T MRI. However, 7 T MRI provides increased sensitivity for detection due to enhanced susceptibility effects [63], as exemplified in Fig. 4.

#### Central vein sign

Another emerging MS biomarker—which was also integrated into the diagnostic criteria in 2024—is the central vein sign [47]. MS is characterized by perivenous pathology marked by the accumulation of inflammatory cells surrounding venules. These small veins can best be visualized in the center of MS lesions on susceptibility‐weighted sequences. Studies have demonstrated that the central vein is present in >85% of MS lesions and a minority of cases in other neuroinflammatory diseases. Therefore, the central vein sign constitutes a diagnostic tool to differentiate MS from other related conditions [48]. In 7 T MRI, veins are better delineated due to their paramagnetic properties from deoxygenated blood. Studies have shown that 7 T MRI outperforms 3 T MRI in the detection of a central vein with a 100% sensitivity and specificity for MS [64, 65]. As shown in Fig. 4, central veins are clearly depicted.

#### Leptomeningeal enhancement

Typically, MS lesions are located in proximity to the CSF, around the ventricles and the cerebral cortex. It has been proposed that meningeal inflammation and CSF‐mediated factors may play a key role in the pathogenesis of MS [66]. Visualizing meningeal inflammation has been a challenge on lower magnetic field strengths. Using gadolinium‐enhanced FLAIR imaging at 7 T, leptomeningeal enhancement was observed in 90% of patients with MS in one study. These findings are consistent with observations from postmortem studies. For this reason, 7 T MRI may be useful in diagnosing meningeal pathology [67]. However, leptomeningeal enhancement is not specific for MS and can be seen in other neuroinflammatory diseases as well as non‐inflammatory neurological diseases [68, 69].

### Parkinsonisms

Parkinsonism is an umbrella term for progressive neurodegenerative movement disorders characterized by symptoms, such as rigidity, bradykinesia, and tremors, mainly affecting the elderly population. This category includes various diseases, with Parkinson's disease (PD) being the most common, representing about 80% of all cases. Other forms of parkinsonism include multiple system atrophy (MSA) and progressive supranuclear palsy (PSP). It can be challenging to clinically differentiate PD, PSP, and MSA due to overlapping symptoms [70].

The pathology in PD is characterized by loss of dopaminergic neurons in the substantia nigra pars compacta (SNpc). The SNpc is subdivided into the matrix and five nigrosomes. Nigrosome‐1 is the largest, and it contains the highest concentration of neuromelanin‐rich dopaminergic neurons [71, 72, 73].

The higher spatial resolution and contrast of 7 T enable clearer depiction of small anatomical structures such as the subthalamic nucleus, s, red nucleus, and the basal ganglia. This is particularly beneficial in MRI‐guided treatments such as deep brain stimulation, which targets deep nuclei to manage movement disorders such as PD [74, 75].

Seven Tesla MRI enables more detailed imaging of subregions within the suSN and better visualization of nigrosomes in the SNpc. This improves the detection of abnormal brain architecture in PD [71]. A study on neuromelanin 7T imaging of the SN showed that patients with PD exhibited significantly lower SNpc volumes compared to non‐PD, and PD volume was highly specific and sensitive for differentiating PD from non‐PD and essential tremor [76].

The so‐called swallow tail sign represents the normal appearance of the SN on SWI [77], characterized by a dorsolateral hyperintensity within two hypointense layers, resembling a swallow's tail. The hyperintensity on MRI corresponds to nigrosome‐1 [32, 78]. The loss of the swallow tail sign reflects the degeneration of dopaminergic cells within nigrosome‐1 accompanied by iron accumulation and loss of neuromelanin, and it has been proposed as a diagnostic marker for PD [79]. The absent hyperintensity within the SNpc on 7 T *T*2*‐weighted imaging has been shown to be highly sensitive and specific for PD [78, 80]. This marker can help distinguish PD from other movement disorders. However, patients with PSP and MSA may also exhibit an absent nigrosome‐1 [71].

#### QSM in parkinsonism

QSM is a post‐processing method from the MRI phase images that quantifies iron, calcium, and blood compounds by measuring their effects on local magnetic susceptibility shifts. Using deconvolution, QSM converts magnetic field variations into susceptibility maps. This provides a more accurate measurement of tissue susceptibility compared to other methods [81].

In PD, iron accumulation is linked to the loss of dopamine neurons in the SNp due to oxidative stress resulting in neuronal damage. Studies on 3 T MRI have shown that patients with PD have higher susceptibility values in the SN compared to healthy controls [82, 83]. QSM is a valuable tool for potential early diagnosis and disease monitoring. Additionally, QSM may be useful for assessing treatment efficacy by tracking changes in iron levels within the SN [84].

QSM at 7 T offers significant advantages due to the more pronounced phase shifts at higher magnetic field strengths. This makes it easier to detect variations in magnetic susceptibility, leading to better differentiation between tissues as well as the ability to identify subtle changes in tissue composition. Furthermore, the higher spatial resolution of 7 T MRI provides more accurate susceptibility maps by allowing for more precise mapping of susceptibility variations [85, 86].

The red nucleus, located near the SN, is another region with a high concentration of iron. It is a component of the cerebellar circuitry, often affected in these conditions. Studies using QSM at 3 T found that patients with PSP had significantly higher susceptibility within the red nucleus compared to those with PD, MSA, and healthy controls [87]. Another study could further confirm that the red nucleus shows elevated iron content in PSP, making it a promising marker for differentiating PSP from other parkinsonisms [73].

### Tumors

The increased magnetic field strength of 7 T MRI enables more detailed visualization of fine structures, such as tumor margins, microvasculature, small lesions, microbleeds, and subtle anatomical changes that might be missed at conventional field strengths. This is particularly useful for identifying early signs of tumor growth or recurrence and for detailed mapping of tumor‐associated vasculature [88, 89].

Compared to 3 T, 7 T can provide greater contrast between healthy white matter and glioblastoma tissue, with enhanced visualization of tumor infiltration into adjacent white matter tracts [85] and improved demarcation of tumor boundaries. The improved delineation of tumor borders can help spare more healthy brain tissue. Incorporating 7 T MRI into neurosurgical navigation and radiotherapy treatment planning is technically feasible and safe [90].

SWI and DWI techniques are instrumental in analyzing brain structures such as microbleeds and tumor vasculature, aiding in preoperative tumor grading, tumor microstructure characterization, and visualizing radiation therapy injuries [89].

Glioma diagnosis traditionally requires histopathological analysis, with MRI providing grade estimation based on contrast enhancement, which has limitations. Imaging techniques such as SWI are particularly useful in visualizing neovascularization in malignant gliomas and venous structures and have the potential to improve accuracy. Longitudinal studies using 7 T SWI have tracked tumor vasculature changes during anti‐angiogenic therapy, highlighting changes in brain edema, microhemorrhages, and intratumoral irregularities. SWI is also effective in assessing radiation‐induced injuries [91, 92, 93]. Additionally, 7 T SWI can provide quantification of intratumoral vascular complexity via fractal dimension analysis—correlating with tumor grades—and SWI‐derived local image variance has been linked to tumor grade and IDH status [94].

The ability of DWI to visualize tissue microstructures has been validated [95] for glioma evaluation, with 7 T providing a superior tissue complexity visualization compared to 3 T. This reveals unique tissue information absent in anatomical images. Consistently acquiring high‐quality diffusion data at 7 T is challenging but will support the implementation of comprehensive brain MRI examinations at ultra‐high fields.

### Vascular disorders

#### Vascular MRI imaging techniques

MRI provides an alternative to computed tomography angiography and digital subtraction angiography without ionized radiation [96, 97]. Notably, time‐of‐flight MRI angiography is a contrast agent‐free technique ideal for patients with contraindications for contrast administration such as renal insufficiency or pregnancy [96]. Compared to conventional field strengths, 7 T MRI enables visualization of intracranial vessels in more detail—especially small branches—through higher spatial resolution and longer T1 relaxation of background tissues, which increases the contrast by improving background suppression [98]. Examples of 7 T arterial time‐of‐flight angiography and SWI for vascular imaging are provided in Fig. 2.

#### Atherosclerosis

Intracranial atherosclerosis is defined as atherosclerotic plaques in the large intracranial arteries resulting in increased vessel wall thickness, and it is a common cause of recurrent strokes [99]. With vessel wall MRI, these plaques can be visualized as focal wall thickening within the arterial lumen, showing contrast enhancement primarily at the fibrous cap region. Vessel wall imaging can be improved at 7 T relative to 1.5 and 3 T due to increased signal‐to‐noise ratio and smaller voxels, which are beneficial for detecting subtle pathologies. Moreover, higher magnetic field strength contributes to enhanced CSF suppression, thereby improving image quality in vessel wall imaging by minimizing artifacts arising from the CSF [100, 101, 102].

#### Aneurysm

Intracranial aneurysms are estimated to occur in 0.5%–3% of the population and contribute to 80%–85% of cases of non‐traumatic subarachnoid hemorrhages. Various factors—including aneurysm size, location, and patient age—play roles in determining the risk of rupture [103]. The identification of contrast enhancement within the aneurysm wall has been proposed as an indicator of increased risk of rupture, which can be detected through vessel wall MRI. In addition, contrast enhancement within the wall is a potential marker for assessing the probability of a certain aneurysm having ruptured or the risk of developing vasospasm after rupture. The enhanced spatial resolution provided by 7 T leads to a more accurate characterization of the aneurysm wall in terms of thickness, layers, contrast enhancement, and the ability to detect smaller aneurysms compared to lower magnetic field strengths [104].

#### Vasculitis

Cerebral vasculitis is characterized by inflammation of vessel walls within the central nervous system. Early detection is key to halting the disease's course and preventing irreversible brain damage. Angiographic techniques are used to visualize vasculitis‐related pathology—typically as localized or multifocal segments of stenosis of the intracranial arteries, with a predilection for the middle cerebral artery. In addition, bilateral infarctions across multiple vascular territories may be present [105]. The improved resolution with 7 T imaging offers a significant advantage in the detection of vasculitis, as it heightens the visibility of smaller intracranial vessels [98]. Furthermore, high‐resolution vessel wall imaging may aid in differentiating vasculitis from conditions such as atherosclerosis. A study on 3 T MRI demonstrated different enhancement patterns, characterized by eccentric enhancement in atherosclerotic plaques and concentric enhancement in inflammation, thereby improving diagnostic precision [106].

#### Arteriovenous malformations

Arteriovenous malformations (AVMs) are high‐flow vascular abnormalities where a nidus connects arterial feeders to draining veins, leading to arteriovenous shunts, vessel hypertrophy, and potential complications such as hemorrhage from ruptured flow‐related aneurysms, venous thrombosis, and infarction due to blood bypassing the brain parenchyma [107]. Seven Tesla MRI is comparable to digital subtraction angiography for evaluating AVMs, as it enables the detection of small flow‐related aneurysms (less than 2–3 mm) and provides a detailed characterization of vascular territories for feeding arteries and draining veins [108]. Another technique called 4D flow MRI enables quantitative examination of blood flow within vessels, providing insights into hemodynamics within AVMs and other cerebrovascular diseases with disturbed blood flow, such as Moyamoya disease. Improved resolution is crucial for capturing hemodynamic alterations within small vessels and aneurysms, which previously was a limitation with 4D flow MRI at lower magnetic field strengths [109].

#### Cavernous malformations

Cavernous malformation, also called cavernoma, constitutes 8%–15% of all vascular malformations and is characterized by clusters of dilated, thin‐walled capillaries containing hemosiderin deposits within and surrounding the lesions. Typically, these lesions remain asymptomatic; however, in rare instances, they can cause hemorrhage, particularly when combined with developmental venous anomalies [110]. Due to the paramagnetic properties of hemosiderin, these lesions are visualized best on SWI. With 7 T MRI, the sensitivity to detect cavernomas is significantly improved through increased susceptibility effects, and even microscopic lesions can be visualized [111].

## Functional and metabolic imaging

### fMRI

Task‐based functional MRI (fMRI) is routinely used for presurgical planning to identify eloquent brain regions, such as those governing speech and motor functions [112]. Clinical 7 T MRI enhances this process by offering superior spatial resolution and more precise delineation of functional areas. By improving the accuracy of these maps, 7 T fMRI helps minimize the risk of postoperative deficits in patients undergoing surgery for brain tumors, epilepsy, or vascular malformations, providing a significant clinical advantage over lower field systems.

Another advantage with 7 T MRI is that it can provide layered fMRI, disentangling the activation and inhibition within individual cortical layers. This precision is crucial for understanding the directional flow of information within cortical circuits, as it permits differentiation between feedforward and feedback processes across layers [113]. Although this presently does not hold immediate clinical diagnostic value, it may help us better understand network diseases such as epilepsy and give insight into disrupted communication pathways in disorders, such as depression, schizophrenia, and anxiety. These advances may inform the development of targeted therapeutic strategies in the future, bridging the gap between neuroimaging research and clinical psychiatry.

### Spectroscopy

Proton magnetic resonance spectroscopy is a non‐invasive imaging technique that quantifies brain metabolites in vivo, including *N*‐acetylaspartate (neuronal integrity), creatine (energy metabolism), choline (cell membrane turnover), *myo*‐inositol (glial activation), and neurotransmitters such as glutamate (excitatory) and GABA (inhibitory) [114]. In clinical practice, proton magnetic resonance spectroscopy is routinely applied to evaluate metabolic disorders in children and can detect lactate peaks for distinguishing brain abscesses from other pathologies.

The increased spectral resolution and signal‐to‐noise ratio at 7 T enable precise quantification of low‐concentration metabolites and resolution of overlapping spectral peaks [115]. This is particularly valuable for researching neurological and psychiatric disorders with improved measurements of glutamate and GABA, aiding in the understanding of epilepsy, depression, and schizophrenia. The enhanced resolution also supports regional metabolic assessments in conditions, such as brain tumors, MS, ALS, and AD [115, 116, 117].

### CEST

Metabolic imaging at 7 T MRI, particularly using chemical exchange saturation transfer (CEST) techniques, has shown advanced capabilities for tumor characterization and treatment monitoring. Variants, such as amide‐proton‐transfer CEST, glutamate‐CEST, and glucose‐CEST, leverage the improved frequency dispersion at 7 T to enhance signal specificity, enabling applications such as tumor grading, prediction of treatment efficacy, and identification of genetic mutations such as IDH status and MGMT promoter methylation [118, 119, 120, 121]. Studies have demonstrated that glucose‐based CEST methods provide superior tumor border identification and contrast enhancement without the risks associated with gadolinium agents, showing significant promise for non‐invasive, high‐resolution metabolic imaging at 7 T [122, 123, 124, 125].

### X‐nuclei imaging

Typically, clinical MRI is based on imaging the spin of protons, which are abundant in the human body with its high water content. However, MRI also has the potential to visualize other elements with spin that are crucial for numerous physiological processes, providing opportunities for non‐invasive metabolic imaging without ionizing radiation. This imaging technique is known as X‐nuclei, non‐proton, or multinuclear MRI [126]. Examples of X‐nuclei used in biomedical applications are ^13^C, ^17^O, ^19^F, ^23^Na, and ^31^P [127]. In comparison to ^1^H, X‐nuclei have a much lower abundance in vivo, which limits the signal‐to‐noise ratio, making it an attractive 7 T application [74]. Because different X‐nuclei are involved in distinct physiological activities, X‐nuclei imaging techniques can be used to study a broad spectrum of diseases [127, 128]. Although X‐nuclei imaging shows promise for metabolic imaging, especially at 7 T MRI, it remains thus far mainly a tool for research and better pathophysiological understanding of diseases.

## Future directions

Clearly, clinical 7 T MRI holds significant potential for advancing diagnostic precision and patient care across neurological conditions. To further improve the clinical value, future efforts should build on existing knowledge to define clinical standards in terms of protocols and standard operating procedures for safety management. Establishing a common database on known safe, conditional, and tested implants would be especially valuable. Sharing experiences through international collaborations among early‐adoption sites is also encouraged to establish harmonization for clinical 7 T MRI. This is a necessary step to build a foundation for multicenter trials and the ability to pool and validate data across sites.

Medical professionals and vendors will need to work together with legislators and insurers to help define the clinical and monetary value of the medical advances that 7 T MRI provides in order to correctly reimburse such scans and make them more available in clinical practice.

Additional hardware and software solutions can bring improvements and expand clinical applications. Field monitoring probes can reduce artifacts and improve image quality [129]. Improved head–neck coils and spinal coils can make it possible to image the whole neuroaxis in one session [130]. Future efforts should continue leveraging high‐gradient performance to further use quantitative imaging techniques to probe microstructural properties and gain improved biological tissue specificity [130]—for example, through myelin imaging, QSM, and advanced diffusion imaging. Notably, a head‐only 7 T system equipped with ultra‐high gradients of 200 mT/m and a slew rate of 900 T/m/s has already been developed [130].

Moreover, the technical capabilities of the second‐generation clinical 7 T scanner provide unique opportunities to bridge the mesoscale gap between in vivo imaging and gold‐standard histopathology. Notably, ex vivo MRI at ultra‐high resolution (150 µm, exemplified in Fig. S1) presents opportunities to improve our understanding of underlying pathophysiology, guide targeted advanced histopathological analysis, and identify new treatment targets.

To continue pushing the frontiers of neuroimaging, additional effort should leverage the capabilities of 7 T and higher field strengths (10.5, 11.7, and even 14 T) [113]. From a sustainability standpoint, developing cryogen‐free magnet designs will ensure broader accessibility and environmental responsibility [131].

## Conclusions

Clinical 7 T MRI represents a transformative advancement in neuroimaging, offering unparalleled diagnostic precision. Its application in neurological disorders, such as ALS, MS, dementia, parkinsonism, tumors, and vascular diseases, allows for earlier diagnoses, enhanced lesion characterization, and improved treatment planning. Emerging biomarkers used clinically today—such as the motor band sign, central vein sign, and PRLs—highlight its potential to redefine disease understanding and management. Innovations in hardware, software, and safety protocols are addressing previous limitations and expanding accessibility. Future efforts should focus on standardizing protocols, enabling multicenter collaborations, and integrating 7 T MRI into routine practice through demonstrated cost‐effectiveness. By bridging research and clinical care, 7 T MRI has the potential to reshape the diagnostic and therapeutic landscape in neurology.

## Author contributions

Tobias Granberg initiated the study. Russell Ouellette, Elisabeth de Vries, and Tobias Granberg created figures. Tobias Granberg, Elisabeth de Vries, Caroline Hagbohm, and Russell Ouellette drafted the manuscript. All authors revised the manuscript critically and approved the final version of the manuscript.

## Conflict of interest statement

The authors declare no conflicts of interest.

## Funding information

No specific funding was obtained to perform this study.

## Supporting information




**Fig. S1**. Ex vivo 7 Tesla MRI (isotropic 150 µm resolution) showing extensive subpial (Types III and IV) cortical multiple sclerosis lesions.

**Supplemental video**. In vivo clinical 7 Tesla MRI showing multiple sclerosis white matter and gray matter lesions using axial 2D *T*2*‐weighted gradient‐recalled echo with a resolution of 0.3 × 0.3 × 0.9 mm^3^.

## Data Availability

Data sharing is not applicable to this article as no datasets were generated or analyzed during the current study.
