# Supplementary figures and images for: Clinical 7 Tesla magnetic resonance imaging: Impact and patient value in neurological disorders
Source: J Intern Med. 2025 Jan 8;297(3):244–61. doi: 10.1111/joim.20059 (PMC11846079; doi:10.1111/joim.20059)

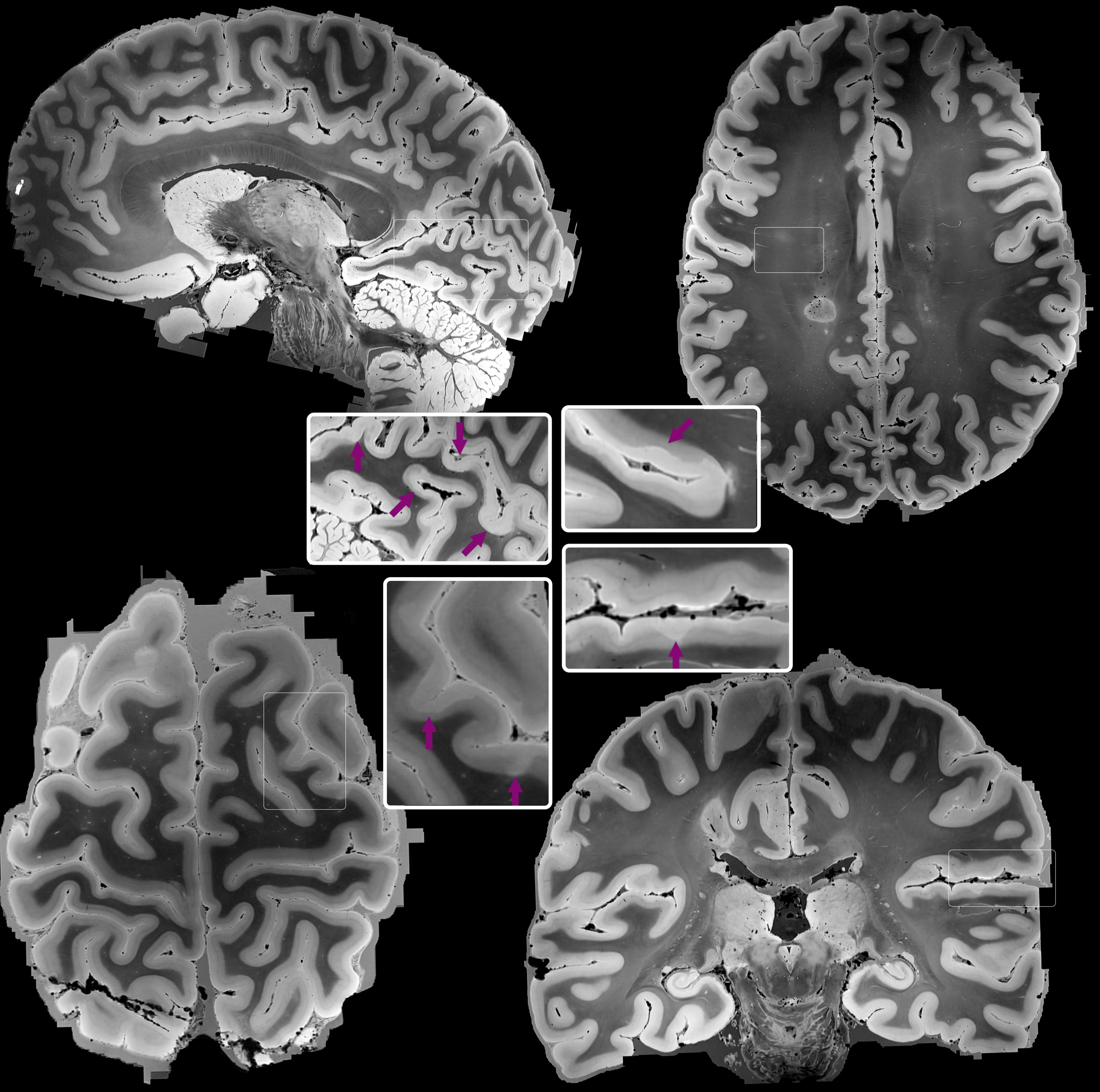

Supplement: Supplementary file 1 — Fig. S1. Ex vivo 7 Tesla MRI (isotropic 150 µm resolution) showing extensive subpial (Types III and IV) cortical multiple sclerosis lesions. [file JOIM-297-244-s002.jpg]
